# Advances of interventional radiology in treatment of hepatobiliary diseases in Iran

**Published:** 2011-07-01

**Authors:** Hossein Ghanaati, Kavous Firouznia, Amir Hossein Jalali, Madjid Shakiba

**Affiliations:** 1 Advanced Diagnostic and Interventional Radiology Research Center (ADIR), Medical Imaging Center, Imam Khomeini Hospital, Tehran University of Medical Sciences, Tehran, IR Iran

**Keywords:** Interventional radiology, Biopsy, Transjugular intrahepatic, Portasystemic shunt, Hepatocellular carcinoma

## Abstract

Interventional radiologists are physicians who specialize in minimally invasive targeted therapies, offering the most in-depth knowledge of the less invasive therapies that are available and diagnostic and clinical experience across all specialties. Interventional radiologists offer treatments for hepatobiliary diseases without significant side effects or damage to the adjacent normal tissue. We briefly introduce some of the interventional procedures in gastroenterology.

## 1. Introduction

Recently, medical science has advanced rapidly, and new interventional techniques for the diagnosis and treatment of diseases have been introduced. Interventional radiology is a safe, less invasive therapy with less recovery time compared with open surgery. Gastroenterology is a specialty that is linked to interventional radiology. Fortunately, there have been significant advances in innovative radiology in the diagnosis and treatment of gastroenterology diseases in Iran. Patients who have suffered hepatobiliary problems benefit from interventional radiology (IR) techniques. First-line modern imaging modalities, such as duplex ultrasonography, 3 and 4 phasic CT scan, and high-resolution MRI, have revolutionized the diagnosis and management of hepatobiliary diseases. Pioneers in this field have performed IR procedures, such as transjugular intrahepatic portosystemic shunt (TIPS), transjugular liver biopsy (TJLB), biliary stent, and embolotherapy, for the hepatobiliary system. Recently, interventional radiologists, in cooperation with gastrohepatologists, have played an important role in developing this field of medicine. We briefly introduce some of the procedures that have been performed in Iran.

## 2. Image-guided liver biopsy 

The safety and efficacy of imaging-guided percutaneous biopsies have been well documented, and because percutaneous intervention is less invasive and more cost-effective than surgery, the number of radiological procedures is continually increasing. CT-guided biopsy is the preferred modality for guidance in many interventional techniques, due to its ability to provide images from different areas. Until recently, liver biopsies were performed without guidance, but now these procedures are being performed as an image guided technique in Iran.

### 2. 1. Navigation

In patients who have platelet dysfunction or low platelet count and in those who have altered Protrombine Time, there is higher risk of bleeding after organ biopsy, especially in the liver. We use CT fluoroscopy to biopsy small lesions. We have published our results regarding fluoroscopic CT (FCT) and have compared it with conventional CT scan. The success rate of FCT (92%), however, was significantly (P = 0.019) higher than that of CCT (65%) for liver mass biopsies. The mean ± SD procedure time for the FCT group was 200 ± 80 sec (20-400) and 420 ± 260 sec (605-800) for the CCT group (P = 0.001) [1].

### 2. 2. Hemorrhage control

We use the coaxial needle (TSK Laboratory/Japan) exclusively for liver biopsy, and if we encounter significant hemorrhage, we inject 2 cc of gel-foam particles, properly mixed with normal saline, via a coaxial needle inside the liver biopsy tract, which we have observed is beneficial for major hemorrhages. In 55 cases in our center, there were no hemodynamic changes that indicated hemorrhage during recovery.

## 3. Transjugular liver biopsy in patients with hemophilia

Most hemophilia patients who were exposed to clotting factor concentrates prior to the introduction of effective viral inactivation techniques have been exposed to blood-borne hepatitis viruses. Hepatitis is observed in 40% of hemophilic patients and is a major cause of death among these patients. Liver biopsy is a necessary component of the evaluation of patients with chronic liver disease, particularly in patients who are infected with HCV. A liver biopsy is important to help determine the necessity of interferon therapy. Transjugular liver biopsy (TJLB) was first performed in Iran in 2001 by Ghanaati et al. In our opinion, the transjugular route that is associated with factor replacement is preferred in reducing the risks of liver biopsy in this population. All transjugular liver biopsies were performed by an interventional radiologist. Although it was safe and clinically useful, the procedure was initially performed by applying suction through the biopsy needle, effecting such complications as hematoma of the neck at the puncture point, hematoma at the level of the brachial plexus, right hypochondriac pain, and abdominal discomfort. Outpatient transjugular liver biopsy is a safe option in patients with hemophilia, avoiding costly and unnecessary hospital admissions. Patients with hemophilia may undergo an outpatient liver biopsy as part of their management of hepatitis C infection [2][3][4]. We performed TJLB for the first time in Iran in 12 patients with congenital bleeding disorders (CBDs) who experienced chronic HCV infection and elevated liver enzymes to determine its safety and efficacy. We used the modified Ross needle, with 100% transjugular access rate to the hepatic veins and 92% success rate in obtaining tissue. The specimens that were obtained were satisfactory, but only 54.5% could be diagnosed histopathologically. Mild hepatitis occurred in four patients (36.4%), moderate hepatitis in 5 (45.4%), and extended fibrosis or cirrhosis in 2 (18.2%). There were 2 procedure related complications (16.6%) [5].

## 4. Transjugular intrahepatic portosystemic shunt (TIPS)

The transjugular intrahepatic portosystemic shunt (TIPS) is a percutaneous intervention that creates a portosystemic shunt, which was performed for the first time in Iran in 2001 by Ghanaati et al. In this method, the shunt is established directly inside the liver by connecting one main portal branch with a large hepatic vein. The parenchymal tract is kept open using an expandable stent. Based on the diameter of the stent, various amounts of portal blood are diverted into systemic circulation, resulting in decompression of portal hypertension. TIPS is a relatively simple procedure that is usually completed in 1 hour. Significant anatomical distortion of the liver or abnormalities of the portal vein, including portal vein thrombosis, can lengthen the procedure. Indications include prevention of variceal bleeding, acute bleeding of esophageal varices that is refractory to medical therapies, esophageal variceal rebleeding, bleeding from gastric varices, prevention of bleeding from portal hypertensive gastropathy and gastric antral vascular ectasia, ascites due to cirrhosis, refractory hepatic hydrothorax, hepatorenal syndrome, Budd-Chiari syndrome, veno-occlusive disease, sinusoidal obstruction syndrome, and hepatopulmonary syndrome [6][7].

Absolute contraindications include primary prevention of variceal bleeding, congestive heart failure, multiple hepatic cysts, uncontrolled systemic infection or sepsis, unrelieved biliary obstruction, and severe pulmonary hypertension. Relative contraindications include hepatoma, obstruction of all hepatic veins, portal vein thrombosis, thrombocytopenia of less than 20,000/cm(3), severe coagulopathy, and moderate pulmonary hypertension. Common risks and complications include minor pain, bruising and infection from the IV cannula, pain or discomfort at the puncture site, bleeding or bruising, failure of local anesthetics, nerve damage (usually temporary), pain, and mild encephalopathy (usually temporary). Less common risks and complications consist of infection; damage to the surrounding structures, such as blood vessels, organs, and muscles; excessive bleeding from the liver; an allergy to the injected drug; and a fast or irregular heart beat. Rare risks and complications include rupture of a blood vessel, liver failure, severe encephalopathy (brain intoxication) and seizures, and cardiac arrest due to local anesthetic toxicity.

### 4.1. Technical success and patient survival

The success rate with TIPS for decompression of the portal vein has exceeded 90% in most series [6][7].

## 5. Intravascular treatment of hepatic hemangioma

Hepatic hemangioma is a common benign disease. Most such patients are asymptomatic and do require intervention, but several are symptomatic and are traditionally treated by surgery. In the past decade, interventional treatment of hemangioma, especially large hemangioma, has been effective and is less invasive than surgery or radiation therapy. Surgery, which carries a high risk of complications, requires more recovery time than transarterial embolization. Radiation therapy is effective for hemangiomas with a diameter of less than 5 cm, but for gigantic hemangioma, the therapeutic effect is very limited. In addition, many patients cannot tolerate the large radiation dose that is administered. Polyvinyl alcohol (PVA), ethanol, and lipiodol (LP) emulsions are often used as embolizing agents for transarterial embolization. However, PVA can lead to complications, such as pain [8]. We embolized 23 patients with liver hemangioma. No major complications, such as hepatic failure and abscess formation, were observed. The majority of our cases (87%) experienced significant relief of symptoms.

## 6. Metallic stenting in the management of malignant obstructive jaundice

Malignant obstructive jaundice has a poor prognosis, limiting the patient's lifestyle and survival. Most patients will die in the first 6 months after diagnosis. There is no curative therapy for the disease, but stenting and drainage of the biliary system are safe palliative methods with few complications. This is now a reliable method for palliation of patients with obstructive malignant jaundice, which is superior to plastic endoprosthesis, considering patency rate, quality of life, complication rate, and cost. Percutaneous biliary stenting is a safe procedure with few technical complications and a high success rate of palliation for patients with malignant biliary jaundice. Early complications are primarily managed conservatively, and most deaths are caused by systemic effects of the malignant disease. It is a new procedure in our country, initiated by Ghanaati et al. and results in 50 patients were published in 2010. Such academic reports may be used to improve this field [9].

## 7. Interventional procedures for HCC

Hepatocellular carcinoma (HCC) is one of the most frequent primary malignant tumors in the world, with a poor prognosis and a low response rate, and it is the fourth leading cause of cancer related death worldwide [10]. Unfortunately, surgical therapies are suitable for 20% of patients; those who are not eligible for surgery should receive undergo interventional therapy. The management of HCC patients requires cooperation between internists, gastroenterologists, oncologists, surgeons, and interventional radiologists, and health service providers should be asked to participate in this multidisciplinary management. In the past decade, various interventional procedures have been employed for local control of HCC, including transcatheter arterial chemoembolization (TACE) and many tumor ablation techniques, such as percutaneous ethanol injection (PEI), radiofrequency ablation (RFA), percutaneous microwave coagulation therapy (PMC), laser-induced interstitial thermotherapy (LITT), cryoablation, and acetic acid injection. Based on the development of new technologies in imaging and drug delivery, it is likely that patients with HCC will be treated with combination therapies to improve survival [11]. At present, these techniques have been introduced to academic radiological centers of Iran.
